# Effects of work-family conflict, social support and burnout on job satisfaction among primary care physicians in Huaihai economic zone

**DOI:** 10.3389/fpsyt.2024.1439636

**Published:** 2024-08-29

**Authors:** Zongliang Wen, Shenqin Wu, Long Bai, Xu Jintao, Yun Zhao, Jinhua Fang, Hamdi Abdirizak Jama

**Affiliations:** ^1^ School of Management, Xuzhou Medical University, Xuzhou, China; ^2^ School of Public Health, Xuzhou Medical University, Xuzhou, China; ^3^ Affiliated Hospital of Xuzhou Medical University, Xuzhou, China; ^4^ Record Room, The Sixth People’s Hospital of Nantong, Nantong, China

**Keywords:** work–family conflict, job satisfaction, burnout, social support, primary care physicians

## Abstract

**Background:**

Primary care physicians (PCPs) are doctors in primary health care institutions, namely village clinics, township health centers and community health service centers (stations) who are the main providers of primary health care services in primary health care settings. Improving the overall health status of the population requires the support of a large number of primary care physicians; however, the job satisfaction of this group has not been sufficiently emphasized and recognized.

**Objective:**

The purpose of this study was to examine the effects of primary care physicians’ work-family conflict on their job satisfaction, as well as the mediating role of burnout and the moderating role of social support.

**Methods:**

This cross-sectional study was conducted from February 2023 to March 2023. Participants were 749 primary care physicians from four cities of Xuzhou, Linyi, Huaibei, and Shangqiu in the Huaihai Economic Zone of China. SPSS statistical analysis was used to evaluate the relationship between work-family conflict, social support, burnout and job satisfaction among medical workers.

**Results:**

Work–family conflict had a significant negative effect on job satisfaction (*β* = −0.36, *p*< 0.001), after adding burnout in the model, work–family conflict also negatively predicted job satisfaction (*β* = −0.32, *p*< 0.001). Social support had a moderating effect on the direct effect of burnout on job satisfaction (*β* = 0.00, *t* = 2.66, *p*< 0.01, 95%*CI* [0.001, 0.007]), the predictive effect of burnout on job satisfaction at high level of social support (*β* = −0.45, *p*< 0.001) was higher than a low level of social support (*β* = −0.33, *p*< 0.001).

**Conclusions:**

This study demonstrated the negative impact of work-family conflict on primary care physicians’ job satisfaction, as well as the mediating role of burnout and the moderating role of social support on burnout and job satisfaction, which are important for improving primary care physicians’ job satisfaction and enhancing the quality of primary care in the future.

## 1 Introduction

Primary care physicians (PCPs) are doctors in primary health care institutions, namely village clinics, township health centers and community health service centers (stations), including specialists, general practitioners, rural doctors and other clinical front-line personnel (1). Primary care physicians in this paper exclude public health practitioners, nurses, pharmacists, administrators, etc. who do not directly provide medical services. Village doctors, community doctors and other primary care physicians are responsible for the prevention and treatment of common, frequent and chronic diseases among urban and rural residents, as well as public health and family contracting services, and are a vital part of China’s medical and health care workforce. Primary care physicians are the “gatekeepers” of residents’ health, and the grassroots first diagnosis is the first line of defence in preventing and treating diseases. China’s national conditions are complex, with large regional differences, different urban and rural development, and the gap between the rich and the poor still existing. The National Health Commission of the People’s Republic of China hopes to build a stable three-tiered healthcare system similar to that of the distribution of tiers in society as a whole, and this stable system requires that the grassroots level be thick enough to support the health needs of the majority of urban and rural residents. At the grassroots communities, many patients are unable to receive good medical help due to various reasons, resulting in ‘minor illnesses becoming major illnesses, and major illnesses becoming difficult illnesses. The goal of primary care physician is to help primary care patients spend less money and time to treat their illnesses and relieve their pain by combining their own medical level and capabilities.

With the reform and development of primary medical care in China, the job satisfaction of primary care physicians has become a topic of concern. As the reform of China’s healthcare system continues to deepen, people’s health awareness is increasing, and the demand for high-quality healthcare services is also increasing (2). Job satisfaction is an emotional response perceived by employees from both psychological and physiological perspectives to the work itself and the work environment, that is, employees’ sense of fulfillment, and an essential indicator for evaluating individual work achievement and work value (3, 4). Job satisfaction among medical professionals is a generalized attitude that is the result of a combination of factors in the workplace (5). Healthcare professionals with high job satisfaction are able to provide more and higher quality healthcare services and have a stronger sense of belonging to the hospital (6). On the contrary, low job satisfaction reduces healthcare workers’ motivation and enthusiasm for work, and also negatively affects patient satisfaction (7).

In China, the overall level of job satisfaction among doctors is low, which negatively affects the process of healthcare delivery. Primary care physicians in primary health care organizations work long hours. People aged between 31 and 40 years old are particularly inclined to spend more time and energy on their work, and in most cases, long working hours lead to more family life conflicts and job dissatisfaction, which may lead to a decrease in job satisfaction (8). Numerous studies have shown that there is a correlation between doctors’ job satisfaction and the level of medical services provided, and between doctors’ turnover intentions and job burnout (9–11). It is essential to improve the job satisfaction of doctors, which affects not only their own health problems and careers, but also patient satisfaction with health care services and the effectiveness of primary health care as a whole (12–14). Therefore, it is significant to identify the factors affecting the job satisfaction of primary care physicians and take effective measures to intervene to improve the quality and efficiency of primary care services.

### 1.1 Work-family conflict and job satisfaction

Work-family conflict has been defined as a form of inter-role conflict that occurs when an employee’s work interferes with his or her family life and occurs when it is difficult to balance the stresses of work and family life (15). There are three main forms of work-family conflict. The first type is time-based conflict: Conflicts that arise as a result of various roles taking up each other’s time, making it difficult for individuals to meet role expectations from work and family within the same time frame. The second type is stress-based conflict, in which the tension and psychological stress experienced by an individual in one role affects performance in another role. The third type is behavior-based conflict, in which an individual performs one role at work or at home in a way that is incompatible with the other role (16, 17). Family-work role conflict is divided into two dimensions. One is the intrusion of work into the family, where the individual invests so much time and energy in work that the demands of family roles and functions are not well met. The other dimension is the intrusion of family into work, where excessive family burdens interfere with work engagement and the fulfillment of work tasks (18, 19). There is a large body of research on the gender-specific impact of work-family conflict, which has found that family responsibilities are the greatest obstacle for female physicians, and that women who sacrifice work time/income to meet family obligations and expectations face greater barriers to career advancement (20). Female health professionals are more likely to experience work-family conflict due to work and family responsibilities (21). Work-family conflict affects women not only by limiting their opportunities to assume leadership positions in professional and specialized activities (22). Primary care physicians are a unique group. They are in the front line of primary care, with long working hours, high work pressure, poor benefits, poor working conditions, and insufficient opportunities for promotion, and are prone to negative emotions. Exploring the conflict between work and family among primary care physicians has essential research value. In the perception and influence of traditional Chinese culture, family is more important to Chinese people. When individuals experience work-family conflict, they tend to perceive a low quality of family life and low satisfaction (23).

Job satisfaction is a subjective psychological response induced by work and is important for motivating employees and improving organizational performance (24). It was found that work-family conflict has a significant negative impact on job satisfaction. Overall, work-family conflict had a greater impact on family conflict, while family-work conflict had a lesser impact on job satisfaction. Work-family relationship had a significant effect on job and life satisfaction (23). Compared with men, female doctors delayed starting a family, required fertility treatments more frequently, had more family responsibilities, assumed fewer academic and leadership roles, earned less, and were less satisfied with work-family balance (25). Researchers have explained the negative impact of work-family conflict on life satisfaction in terms of resources, arguing that work-family conflict, as a role stressor, continually depletes an individual’s resources, which, in turn, affects the individual’s life satisfaction (26). For a long time, researchers and workplace organizations have been focusing on the interference between work and family roles. Individuals find it difficult to balance work and family responsibilities (27), which can create work-family conflict. Working in high workload, stressful and risky environments, their job satisfaction is low and declining (28). Satisfaction is affected by a variety of work-related factors, and it is difficult for individuals to find the perfect balance between work and family responsibilities and expectations, and work-related unpredictability can lead to complexities in both work and personal life that can affect long-term career satisfaction (29). Based on the above studies, we hypothesized that work-family conflict among primary care physicians leads to job satisfaction.

### 1.2 Burnout as a mediating role

Burnout is a long-term response to chronic emotional and interpersonal stressors at work, defined by the three dimensions of exhaustion, cynicism, and incompetence (30). Burnout not only affects the individual, but also the physician’s family. The mediating role of social support in the relationship between physician burnout and professional behavior, the immediate work environment, and the health care system as a whole (33). In work-family conflict, role pressures from the work and family domains are in some ways contradictory, and insufficient participation in either the family role or the work role can make participation in either the work role or the family role more difficult (31). Research has shown that early career professionals have the highest rate of work-life conflict and the greatest difficulty in resolving work-life conflict. Career choice satisfaction is lowest early in a career and rises with age (32). In a study of U.S. surgeons, Lacey found that doctors were concerned about child care; disrupted family activities; missed family dinners; and lacked time with their spouse or partner - four aspects of the work-family conflict problem - were among the leading causes of One of the causes of burnout, which is more likely to occur in two professional families or when the spouse is also a doctor, is highest when a surgeon is married to a surgeon. We can deeply appreciate that when two people are both burdened with demanding careers, the stress can bind them together, sap their energy and enthusiasm, and wear them down (33).

Research has shown that work-family conflict is significantly and positively associated with burnout (34). Work-family conflict appears to be prevalent among physicians and is a potential predictor of burnout (35). A survey of doctors of all ages found that early-career doctors had the lowest satisfaction with their overall career choice (as a doctor), the highest frequency of work-family conflict, and the highest rate of depersonalization. Job burnout is a psychological state that results in negative emotions and pessimistic attitudes due to chronic stress, emotional exhaustion, and lack of work resources (36). Personal/internal factors affecting job burnout and job satisfaction include positive and negative moods/emotions and subjective well-being (37). Job burnout significantly affects job satisfaction. Burnout was significantly negatively correlated with job satisfaction. Mid-career doctors work longer hours, take more overnight phone calls, have the lowest levels of satisfaction with career choices and work-life balance, have the highest rates of emotional exhaustion and burnout, and work long hours under significant job stress that may limit their ability to effectively fulfill their work and family roles (38). Medical professionals have a higher incidence of burnout than other occupations, and burnout is prevalent among general practitioners (39). Burnout reduces an individual’s commitment and enthusiasm for their work, which in turn affects job satisfaction. Therefore, we hypothesized that burnout among primary care physician plays a direct mediating role between work-family conflict and job satisfaction.

### 1.3 Social support as a moderating role

Social support has been found to be a multidimensional and broad structure that promotes the psychological development of individuals. Social support can provide a strong protective function for the healthy development of an individual, reduce the impact of negative events, and promote the maintenance of positive attitudes (40). Understanding social support is an in-depth development of social support. Understanding social support can alleviate an individual’s negative emotions and improve the individual’s sense of well-being in life. When individuals feel more social support, they will have more resources and the ability to face difficult situations in life (41). The ability to understand social support has a buffering effect, which can effectively deal with the negative effects of adverse events, help people gradually recover emotionally from trauma, and have the function of buffering the stress and adverse psychological state brought about by adverse events (42). Social support plays an important role in managing stress, preventing burnout, and thus regulating personal well-being (43). According to the main effects model of social support, social support is beneficial for mental health and can increase well-being and satisfaction, and a lack of support from family, friends, and others can reduce people’s satisfaction (44). The main effects model suggests that social support has a generalized gain effect. Social support can help individuals improve their overall health. The buffering model suggests that social support acts as a buffer through an individual’s internal cognitive system, buffering the effects of stressful events on an individual’s health and protecting the individual from the effects of stress. These two models reflect the function of social support in maintaining health and in prevention and treatment (45).

The aforementioned study found that both affective and instrumental social support moderated the effects of cynicism and emotional exhaustion on job satisfaction, with higher job satisfaction scores in the high support condition (46). Based on existing research and relevant theoretical foundations, we hypothesized that social support plays a moderating role between burnout and job satisfaction.

Nationally, there is little research on examining whether work-family conflict causes burnout in primary care physicians and will reduce job satisfaction and the effect of social support on the relationship between burnout and job satisfaction. Therefore this study formulated three hypotheses to examine the associations between work-family conflict, job satisfaction, burnout and social support.

Hypothesis 1: There is a negative correlation between work-family conflict and job satisfaction of primary care physicians.

Hypothesis 2: Primary care physicians’ burnout will play a mediating role between work-family conflict and job satisfaction.

Hypothesis 3: Social support may play a moderating role in burnout and job satisfaction of primary care physicians.

**Figure 1:**
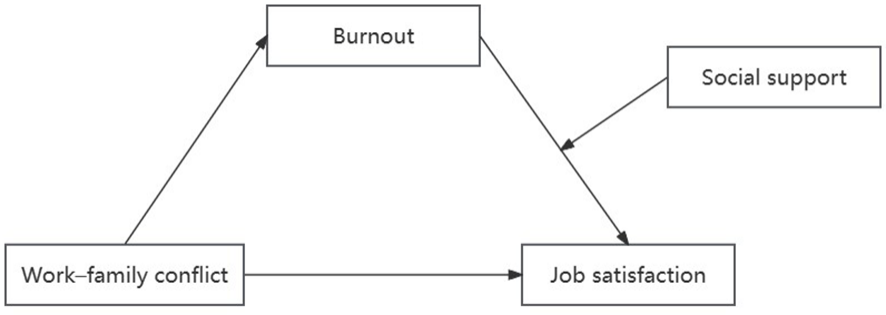
The proposed moderated mediation model of the mediating role of burnout on work-family conflict and job satisfaction and the moderating role of social support on the relationship between burnout and job satisfaction.

The proposed hypothetical model is illustrated in **Figure 1**.

## 2 Materials and methods

### 2.1 Participants, procedure, and ethics statement

The Huaihai Economic Zone is the intersection of four provinces: Jiangsu, Anhui, Shandong, and Henan. The Huaihai Economic Zone’s geographic location is important as part of China’s strategic plan to promote the integrated development of the Yangtze River Delta, which has a large population and arduous healthcare tasks, and there is a strong correlation between primary care physicians’ mental health and the quality of the healthcare services they provide. There are significant differences in the level of economic development of many cities in the Huaihai Economic Zone, ranging from developed cities to medium and more backward ones, reflecting the imbalance of economic development within the region, as well as the diversity and complexity of China’s overall economic development, which is representative for China. Therefore, we chose the study population in the region with the aim of exploring the current situation of primary care physicians.

From February 2023 to March 2023, based on the distribution of population and primary medical staff, a cross-sectional survey of simple random sampling was conducted in Xuzhou, Linyi, Huaibei and Shangqiu cities in China’s Huaihai Economic Zone through repeated communication with the staff of local health Commission. Medical staff at all primary healthcare institutions in the four urban jurisdictions were eligible to participate in the study. To collect the data, we used a self-compiled questionnaire that included the Minnesota Satisfaction Questionnaire (MSQ), the Maslach Burnout Inventory-General Survey (MBI-GS), the Work-Family Conflict Scale (WFCS), the Perceptive Social Support Scale (PSSS), and self-compiled demographic questions. The completion time of the verification questionnaire is about 20 minutes. Staff participating in the survey were informed about the purpose and significance of the study and volunteered to participate. Questionnaires were distributed using the medium of Questionstar and were deleted if they had routine answers (all options were the same) or if there was too much missing data. A total of 796 questionnaires were received, after a preliminary examination of the data, 47 questionnaires were excluded, and 749 primary care physicians were included. This study follows the Declaration of Helsinki as amended in 1989 and was authorized by the Ethics Committee of Xuzhou Medical University (ID: 225281).

### 2.2 Baseline characteristics of the primary medical staff

We collected baseline characteristics of primary medical staff, which include age (≤40, 41–50 and ≥51), sex (female and male), education background (junior college and below, bachelor’s degree and above), technical title (no title, primary title, middle title, vice-senior title, or above), salary (≤3,000 yuan (≤US $415.4), 3,001–5,000 yuan (US $415.4–$692.3) and >5,000 yuan (>US$692.3)], staffing of public institution(yes or no).

### 2.3 Assessment of work family conflict

Work-family conflict assessment was assessed using the Chinese version of the Multidimensional Work-family Conflict Scale (WFCS) (47). The WFCS includes 18 restrictions, including work-to-family interference (WIF) and family-to-work interference (FIW). WIF refers to the negative impact of work-related requirements and obligations on family life. FIW means that conflicts caused by family obligations can interfere with personal work. Both subscales include conflicts of time, stress, and behavior. Work-family conflict was evaluated using a 5-point Likert scale, ranging from 1 (highly disagree) to 5 (highly agree). In this study, the Cronbach’s alpha coefficient for the WFC in this study was 0.958.

### 2.4 Assessment of job satisfaction

Job satisfaction was assessed using the Minnesota Satisfaction Scale (MSQ) (48), a short scale consisting of three subscales of intrinsic, extrinsic, and general satisfaction (e.g., “How satisfied are you with being able to stay busy?”). It is characterized by a complete measurement of the integrity and dimension of job satisfaction. Participants rated each item on a five-point Likert scale (1 = very dissatisfied; 5 = very satisfied), the higher the score, the higher the doctor’s job satisfaction. The Cronbach’s alpha coefficient of MSQ in this study was 0.969.

### 2.5 Assessment of burnout

The burnout of primary care physicians was evaluated with the Chinese version of the Maslach Burnout Inventory-General Survey (MBI-GS) (49). The scale includes three subscales: emotional exhaustion, depersonalization, and low personal achievement (reverse score). The response was set to a 7-Likert score, ranging from 0 (never) to 6 (daily). Emotional exhaustion and depersonalization are scored on a positive scale, meaning the higher the score, the more severe the burnout. Conversely, low personal achievement was scored in reverse, meaning the lower the score, the worse the burnout. These three items are used to assess the individual’s emotional responses, perceptions, feelings and attitudes to work induced by work stress. The Cronbach’s alpha coefficient for the MBI-GS was 0.867.

### 2.6 Assessment of social support

Social support adopted the Chinese version of the perceived Social Support Scale that emphasizes individual self-understanding and self-feeling (PSSS) (50), revised PSSS, changed other support to relatives, colleagues, and leaders, respectively measured the degree of support from various social support sources perceived by individuals, and reflected the total degree of social support felt by individuals with a total score. The Social Support Scale (PSSS) consists of 12 items and uses 3 subscales to assess doctors’ perceived level of social support:(1) Family support (4 items, such as “My family can help me concretely”); (2) Support from friends (four items, such as “My friends can really help me”); (3) Other support (four items, e.g., “When I have a problem, someone will be there for me”). Participants followed a 7-point Likert scale (1 = strongly disagree; A score of 7 = complete agreement), with a high score indicating a high level of support from others. The Cronbach’s alpha coefficient of PSSS is 0.970.

### 2.7 Statistical analyses

We used SPSS 27.0 to calculate the correlations, descriptive statistics, and Cronbach’α coefficients for each variable in this study. Mediation and moderated mediation analysis were conducted using SPSS PROCESS (51). We used a bootstrapping procedure with 5,000 resamples to assess the unconditional indirect effects, which were considered significant when the 95% bias-corrected and accelerated confidence intervals (95% CI) did not contain zero. A two-tailed P-value of<0.05 was considered statistically significant.

## 3 Results

### 3.1 Preliminary analyses

A total of 749 primary care physicians were enrolled in the sample group. The social demographic characteristics of the respondents are shown in **Table 1**. Among them, women accounted for 61.015%, 84.246% were over 40 years of age, 82.243% had college education or less, and there were the largest number of junior-level titles (391, or 52.203%), which are the most basic technical titles. The largest number of employees (391, or 52.203%) had junior titles, the lowest technical title. 62.884% of the employees earned less than 3,000 yuan.

**Table 1:** Socio-demographic characteristics of investigated subjects.

	Characteristics	Subjects (n = 749)	Proportion (%)	Job Satisfaction(mean ± SD)
Age (years)	≤40	118	15.75	78.33 ± 15.37
	41–50	427	57.01	76.93 ± 15.30
	≥51	204	27.24	79.22 ± 14.91
Sex	Female	292	38.99	76.59 ± 14.37
	Male	457	61.02	78.44 ± 15.49
Education background	Technical secondary school	357	47.66	78.20 ± 14.58
	Junior college	259	34.58	78.08 ± 15.37
	Bachelor	133	17.76	75.71 ± 15.75
Technical title	No title	204	27.24	79 ± 15.06
	primary title	391	52.20	77.83 ± 15.20
	middle title	111	14.82	76.04 ± 14.73
	Above vice-senior Title	43	5.74	78.72 ± 10.62
Salary	<3,000	471	62.88	78.03 ± 14.83
	3,000-5,000	201	26.84	78.07 ± 15.71
	>5,000	77	10.28	71.67 ± 16.04
Staffing of public institution	Yes	310	41.39	74.80 ± 14.51
	No	439	58.61	78.20 ± 15.13

We categorized work-family conflict into three measures of conflict of time, conflict of pressure and conflict of behavior. Social support was categorized into three measures of family support, friends support and other support. Burnout was categorized into three measures of exhaustion, cynicism and inefficiency. The correlation of job satisfaction with work-family conflict, social support, and burnout was evaluated by Pearson correlation analysis in **Table 2**. Correlations for all variables are presented in **Table 3**. Work-family conflict and burnout were negatively correlated with job satisfaction (*r*=−0.376 and −0.243, *p*<0.01), work-family conflict was positively correlated with burnout (*r*= 0.429, *p*< 0.01), and social support was negatively correlated with work-family conflict (*r*=−0.186, *p*<0.01), and positively correlated with job satisfaction (*r*=0.431, *p*<0.01).

**Table 2:** Pearson correlation analysis of the relationship of work-family conflict, social support, and burnout with job satisfaction.

Variable	Item measured	Pearson correlation coefficient with job satisfaction	P value
Work-family conflict	Conflict of time	0.888	<0.001
	Conflict of pressure	0.923	<0.001
	Conflict of behavior	0.862	0.000
Social support	Family support	-0.202	<0.001
	Friends support	-0.248	<0.001
	Other support	-0.269	<0.001
Burnout	Exhaustion	0.577	<0.001
	Cynicism	0.482	<0.001
	Inefficiency	-0.089	0.015

**Table 3:** Pearson’s correlation between variables.

	1.Work–family conflict	2. Job satisfaction	3.Burnout	4.Social support
1.Work–family conflict	−			
2. Job satisfaction	−0.38**	−		
3.Burnout	0.43**	−0.24**	−	
4.Social support	−0.19**	0.43**	0.06	−

**Significant at the 0.01 level (two-tailed).

### 3.2 The mediation analysis

**Figure 2:**
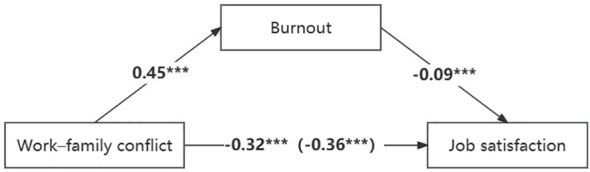
The mediating model. Independent variable: Work-family conflict; Dependent variable: Job satisfaction; Mediating variables: Burnout. ***p<0.001.

A mediation analysis was conducted with model 4 in SPSS PROCESS to examine the mediating effect of burnout between doctors’ work–family conflict and job satisfaction. Because the doctors’ demographic information correlated with the main study variables, we also included these variables (doctors’ age, sex, education background, technical title, salary, and staffing of public institution) as control variables. All the study variables were standardized before the mediation analysis. The results (displayed in **Figure 2** and **Table 4**) showed that, work–family conflict had a significant negative effect on job satisfaction (*β* = −0.36, *p*< 0.001), after adding burnout in the model, work–family conflict also negatively predicted job satisfaction (*β* = −0.32, *p*< 0.001). Work–family conflict also significantly predicted burnout (*β* = 0.45, *p*< 0.001), and burnout negatively predicted job satisfaction (*β* = −0.09, *p*< 0.001). The bias-corrected bootstrap method was used to test the mediating effect. There were 5,000 bootstrap samples run by PROCESS, which indicated that the indirect effect was 0.11, 95% *CI* = [−0.08, 0.01].

**Table 4:** Results of the mediation model of burnout between work–family conflict and job satisfaction.

Dependent variables	Independent variables	R	R^2^	F	β	t	95%CI
Job satisfaction		0.39	0.15	19.10***			
	Sex				−1.41	−1.32	[−3.51,0.69]
	Age				−2.52	−0.66	[−9.99,4.94]
	Education background				−0.44	−0.28	[−3.52,2.64]
	Technical title				1.24	1.60	[−0.29,2.77]
	Salary				0.04	−0.04	[−1.86,1.93]
	Staffing of public institution				2.26	1.54	[−0.62,5.14]
	Work–family conflict				−0.36	−10.97***	[−0.43,−0.30]
Burnout		0.432	0.186	24.231***			
	Sex				−0.59	−0.52	[−2.81,1.63]
	Age				1.98	0.49	[−5.91,9.87]
	Education background				0.83	0.50	[−2.42,4.10]
	Technical title				−0.12	−0.14	[−1.73,1.49]
	Salary				0.25	0.24	[−1.75,2.25]
	Staffing of public institution				−1.07	−0.69	[−4.11,1.98]
	Work–family conflict				0.45	12.85***	[0.38,0.52]
Job satisfaction		0.40	0.16	17.68***			
	Sex				−1.46	−1.37	[−3.55,0.63]
	Age				−2.35	−0.62	[−9.78,5.09]
	Education background				−0.37	−0.23	[−3.44,2.71]
	Technical title				1.23	1.59	[−0.29,2.75]
	Salary				0.58	0.06	[−1.83,1.94]
	Staffing of public institution				2.16	1.48	[−0.71,5.03]
	Burnout				−0.09	−2.58**	[−0.16,−0.02]
	Work–family conflict				−0.32	−8.85***	[−0.39,−0.25]

**p<0.01, ***p<0.001

### 3.3 The moderated mediation analysis

After testing the mediating effect of occupational identity, we tested the moderating effect of social support in the mediation model using Model 59 from SPSS PROCESS. The 95% *CI* does not contain zero means the moderated mediation effects are significant. The *p* value of interaction term burnout × social support is 0.008 less than 0.01, so it exhibited associative stability. The results (see **Table 5**) showed that social support had a moderating effect on the direct effect of burnout on job satisfaction (*β* = 0.00, *t* = 2.66, *p*< 0.01, 95%*CI* [0.001, 0.007]), The independent variable *M* is opposite to the sign of the interaction term, so it has a negative moderating effect. So, the results showed that the moderated mediation effect was significant, doctors’ social support moderated the relationship between burnout and job satisfaction.

**Table 5:** Results of social support moderated mediation model.

Dependent variables	Independent variables	R	R^2^	F	β	t	95%CI
Job satisfaction		0.56	0.32	34.33***			
	Sex				−1.12	−1.16	[−3.00,0.77]
	Age				−2.41	−0.70	[−9.12,4.31]
	Education background				−0.34	−0.24	[−3.11,2.44]
	Technical title				1.26	1.80	[−0.12,2.63]
	Salary				−0.14	−0.16	[−1.85,1.56]
	Staffing of public institution				2.40	1.82	[−0.19,5.00]
	Work–family conflict				−0.21	−6.33	[−0.28,−0.15]
	Burnout				−0.16***	−5.03	[−0.22,−0.10]
	Social support				0.42***	13.02	[0.36,0.49]
	Burnout×Social support				0.00**	2.66	[0.00,0.01]

**p< 0.01,***p< 0.001

**Figures 3:**
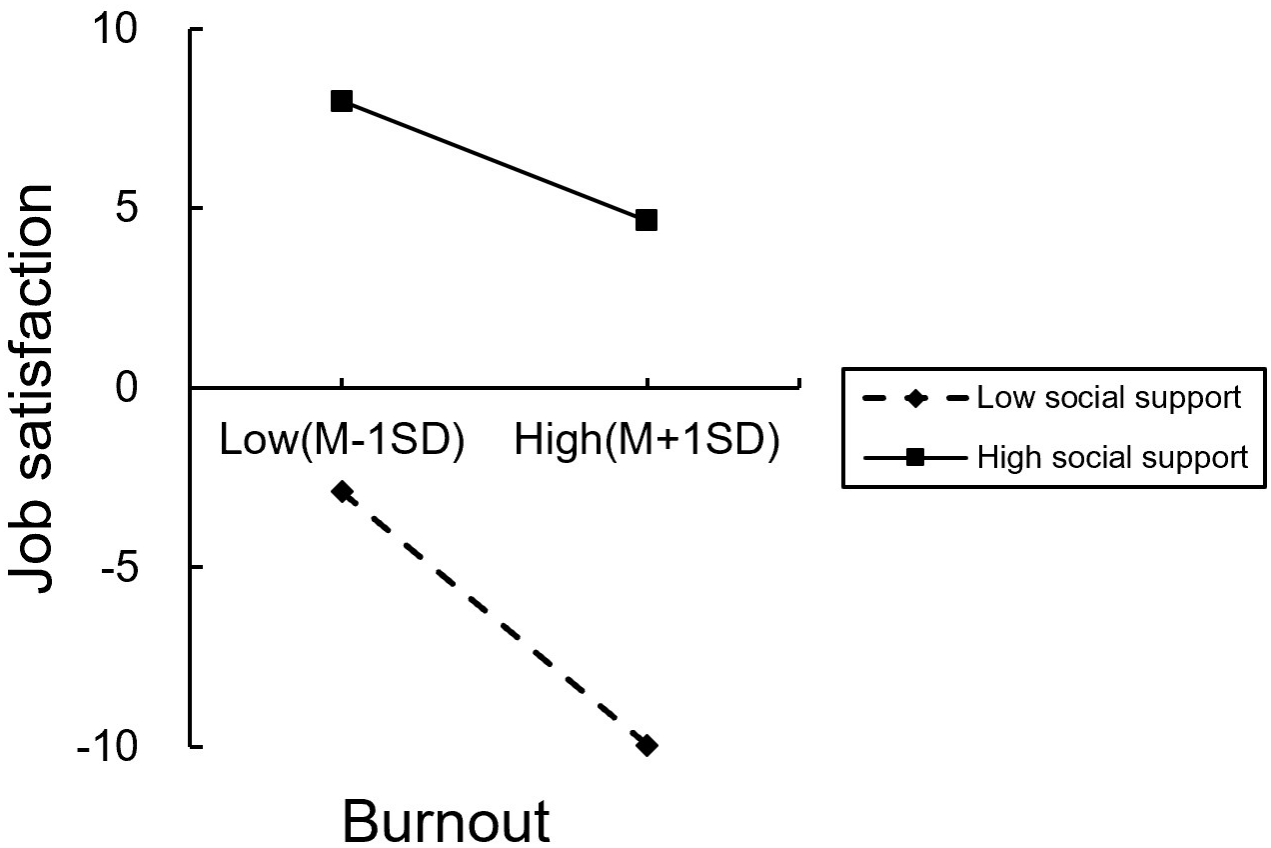
The simple slope test of the moderating effect of social support on the relationship between burnout and job satisfaction.

In order to reveal the essence of the interaction, then we conducted the simple slopes at lower (−1SD) and higher (+1SD) levels of social support (see **Figures 3**), for the moderating effect of social support on the relationship between burnout and job satisfaction, the predictive effect of burnout on job satisfaction at high level of social support (*β* = −0.45, *p*< 0.001)was higher than a low level of social support (*β* = −0.33, *p*< 0.001).

## 4 Discussion

The purpose of this study was to examine the relationship between work-family conflict and job satisfaction among primary care physicians and to further explore whether burnout mediates between them, as well as the moderating role of social support. And to date, this investigation was the first study to explore the moderation mediation mechanism of burnout and social support in the link from work-family conflict to job satisfaction (burnout as the mediating variable and social support as the moderating variable). Our findings demonstrated that the mediation effect of burnout in the pathway from work-family conflict to job satisfaction and the moderating role of social support in the link between burnout and job satisfaction were both significant. The findings also provided new insights into the detail of how social support can play a significant moderating role.

The results of this study showed that work-family conflict among primary care physicians was a negative predictor of their job satisfaction, validating Hypothesis 1, which is consistent with previous findings (52, 53). Increased time and psychological exertion at work decreases the resources available to meet family needs; the more time a person spends at work, the less time is available to meet family needs, and conflict occurs between these two domains due to the lack of resources available to meet the needs of both roles. Conscious harmonization of work and family life, and the development of resources in each area that help to “fit in” and reduce conflict, can help to achieve life balance and satisfaction (27). Role stress from work and family is a major cause of work-family conflict, with employees expressing dissatisfaction with their jobs, whether the job interferes with the family or the family delays the job (25). The demands of work and family life have a significant impact on an individual’s overall well-being and can lead to poorer marital interactions or dissatisfaction (54). Scholars have noted that this is especially true for women compared to men because of their multiple roles as spouses, parents, caregivers, and employees (55). Often, balancing caring for children and aging loved ones with a career (55) affects women’s overall well-being and life satisfaction. According to boundary theory and role theory, employees experiencing work-family conflict have difficulty in making smooth role transitions between work and family life boundaries, and have less control over their work-family life boundaries, which produces a lower sense of boundary control and ultimately leads to a lower sense of satisfaction with one’s life (56).

The results of this study show that burnout among junior doctors partially mediates the relationship between work-family conflict and job satisfaction. Work-family conflict not only negatively predicts job satisfaction directly, but also indirectly affects job satisfaction through the mediating role of burnout, validating Hypothesis 2. Due to time constraints, excessive stress, and competing behavioral expectations, professionals may experience work-family conflict, leading to burnout and even mental illness (57, 58). Heavy workloads combined with inadequate family-friendly workplace policies and practices may be the root cause of work-family conflict and high levels of burnout, poor health, and mental distress (59, 60). Ambiguity at work can have significant costs in terms of decreased creativity, frustration, disappointment, and mental stress, including burnout, which can spill over into one’s family life. This has the potential to reduce one’s satisfaction with one’s career and decrease satisfaction with one’s success in life (30). Higher levels of emotional exhaustion and burnout are associated with lower levels of career efficacy and job satisfaction. Burnout accelerates the depletion of resources, discourages employees from working for the organization, and negatively evaluates their professional achievements, which further leads to negative work attitudes and decreased job satisfaction (61). General practitioners are under-represented, have high work pressure, high work intensity, long daily working hours and often work overtime. Continuous work stress can lead to physical and mental fatigue of general practitioners, leading to burnout, resulting in lower job satisfaction and ultimately affecting the stability of the primary care general practitioner workforce (62).

This study found that social support has a significant moderating effect on the relationship between burnout and job satisfaction. That is, primary care physicians with higher levels of social support could maintain higher levels of job satisfaction at lower levels of burnout compared to primary care physicians with lower levels of social support. Both job satisfaction and social support (emotional and instrumental) measures correlated with burnout scale scores, and increased job flexibility and organizational support had a positive effect on employee satisfaction and performance. Healthcare providers who perceive more social support will have higher levels of understanding and utilization of various social support resources. In this case, they are more likely to take positive action when faced with an adverse event, such as seeking professional help or sharing personal experiences and feelings in social interactions with peers and other supportive members. These positive behaviors help to maintain a positive mood and contribute to a sense of accomplishment and confidence in one’s work, thus increasing one’s life satisfaction (44). Job satisfaction is expected to be lower at levels of high burnout and low social support, which is consistent with previous research that found high cynicism, low emotional social support, and low job satisfaction (46). Doctors with high levels of burnout may be slow to seek help because they may not know how to properly articulate their need for help, and burned-out doctors may become more uncaring toward people and lose their enthusiasm for providing support to their peers, which may naturally cause them to be less socially supportive (63). Psychological interventions are needed to reduce burnout and foster empathy and well-being (64). Doctors work long hours, take fewer vacations, and have less time to engage in social interactions, which will limit the amount of help and support they receive from family, friends, and other members of society. Social support should be strengthened to improve the mental health of doctors and reduce the impact of burnout on their job satisfaction (65).

### 4.1 Implications and limitations

Based on a survey of 749 primary care physicians in Huaihai Economic Zone of China, it is found that burnout is an intermediary between work-family conflict and job satisfaction, and social support has a moderating effect on burnout and job satisfaction. These results have important theoretical and practical significance. First, the mediated model validates the main effect model of social support and broadens the explanatory scope of the social support buffer model. Secondly, the results reveal the current situation of primary care physicians’ job satisfaction and suggest that managers should pay attention to the impact of burnout. Finally, the findings show that social support can alleviate primary care physicians’ burnout and improve their job satisfaction. Therefore, we should consider how to provide more social support for primary care physicians.

This study has several limitations. Our study was a cross-sectional investigation that could not establish causal relationships between variables, and should be followed up with a longitudinal study to understand the patterns and causal relationships of the variables as they evolve over time. Our study was conducted on primary care physicians. Due to the differences between Chinese primary care physicians, including general practitioners, nurses, pharmacists, public health practitioners and health managers, family doctors in the rest of the countries are medical personnel who have gone through three complete stages of medical education, namely, disciplinary education in general medicine, post-graduation education and continuing education, and who conduct annual medical check-ups and follow up on chronic diseases of patients with health insurance. These differences this may have a different impact and do not reflect the situation of general practitioners worldwide. Secondly, the contents of some investigations in this study are relatively sensitive, and there may be report bias in which the respondents are unwilling to provide the real situation. In future studies, we should try to eliminate the concerns of the respondents.

## Data Availability

The raw data supporting the conclusions of this article will be made available by the authors, without undue reservation.
